# A Comparative Study of the Physiological and Socio-Economic Vulnerabilities to Heat Waves of the Population of the Metropolis of Lyon (France) in a Climate Change Context

**DOI:** 10.3390/ijerph17031004

**Published:** 2020-02-05

**Authors:** Lucille Alonso, Florent Renard

**Affiliations:** UMR CNRS 5600 Environment, City and Society, Department of Geography and Spatial Planning University Jean Moulin Lyon 3, Faculty of Geography and Spatial Planning, 69007 Lyon, France

**Keywords:** physiological vulnerability, socio-economic vulnerability, analytic hierarchy process, principal component analysis, heat waves, climate change

## Abstract

Increases in the frequency and intensity of heat waves are direct consequences of global climate change with a higher risk for urban populations due to the urban heat island effect. Reducing urban overheating is a priority, as is identifying the most vulnerable people to establish targeted and coordinated public health policies. There are many ways of understanding the concept of vulnerability and multiple definitions and applications exist in the literature. To date, however, nothing has been done on the territory of this study, the metropolis of Lyon (France). The objective is thus to construct two vulnerability indices: physiological, focusing on the organism’s capacities to respond to heat waves; and socio-economic, based on the social and economic characteristics and capacities of the community. To this end, two complementary methodologies have been implemented: the AHP (Analytic Hierarchy Process) and the PCA (Principal Component Analysis) with Varimax rotation, respectively. The results were then spatialized to the smallest demographic census unit in France. The areas highlighted differed due to conceptual and methodological differences: the highest physiological vulnerabilities are in the center while the socio-economic ones are in the eastern periphery of the urban area. The location of these areas will enable prevention campaigns to be carried out, targeted according to the publics concerned.

## 1. Introduction

**Figure 1:**
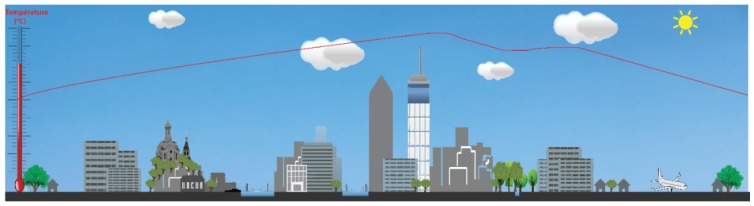
Schematic drawing of a horizontal profile of an urban heat island in the Lyon metropolitan area.

The IPCC climate models on climate change, which integrate scenarios 4.5 and 8.5 of the Representative Concentration Pathways (RCP), predict an increase in annual and seasonal temperatures in France [1,2]. One of the impacts of this climate change is an increase in summer temperatures of 0.5 to 2 °C by 2050 compared to the 1976–2005 baseline period. In addition, there is an increase in heat waves, which have become more severe, more frequent and longer over the past decade [2,3,4]. For example, in Europe, the summer of 2003 had average temperatures 3.5 °C above the normal [5,6]. Thus, temperatures are steadily increasing in most of the world’s major cities: this is due to the impact of climate change combined with the urban heat island effect (UHI) [7,8]. This UHI concept refers to higher observed temperatures in urban areas compared to surrounding rural areas (Figure 1) [9,10]. These two associated phenomena have negative effects on the health of populations by generating heat stress [11,12] which can lead to excess mortality and morbidity [13,14,15,16,17]. In addition, the risk of mortality increases by 1% to 3% for every 1 °C increase [18]. For example, during two weeks in August 2003, temperatures exceeded expected climate variability and resulted in excess mortality due to extreme heat [19]. Thus, at the European level, more than 70,000 deaths were directly related to these heat waves [20]. At the national level, in France, excess mortality was estimated at 20% for the 45–74 year olds, 70% for the 75–94 year old age group, and 120% for people over 94 years old [21]. The heat waves of 2003, 2006 and 2015 are estimated to cause 19,490, 1388 and 3275 deaths respectively (Disaster Database EM-DAT [22]. In also, exposure to at least one heat wave per year doubled between 1974–1983 and 2004–2013 [23].

Furthermore, according to the United Nations [24], the world population is expected to increase by 2 billion people over the next 30 years, from 7.7 billion at present to 9.7 billion in 2050. Moreover, in 1950, only 30% of the world’s population lived in urbanized areas. Today, 54% of the population is affected and in 2050 this figure will rise to 60% [25]. Urban areas are expected to absorb almost all future world population growth [26].

Local public actors are trying to prevent and reduce the human risks potentially generated by these climate and socio-economic changes. In this context, the objective here is to provide a decision support tool based on the assessment and spatialization of two complementary vulnerabilities, based on distinct theoretical and methodological foundations: physiological vulnerability and socio-economic vulnerability. This methodology, adaptable to any territory, is applied here to the Metropolis of the Greater Lyon area. Two complementary vulnerability analyses are thus proposed here, whose concepts, operating mode and results and discussions will be the subject of the text.

## 2. Vulnerabilities: Differences in Approaches and Concepts Applied to the Greater Lyon Area 

Vulnerability is a complicated concept around which several definitions can converge and complement each other without replacing each other. The IPCC refers to the propensity or predisposition to suffer adverse effects [20] and defines vulnerability to climate change as an unbalanced relationship between vulnerability to geophysical, biological and socio-economic systems and the capacity to adapt to or cope with the impacts of climate change [27]. Thus, vulnerability represents the propensity to be adversely affected by a hazard and is said to have three components: sensitivity, exposure and adaptive capacity [28,29,30]. S. Cutter specifies that it is a personal potential for damage that can be related to both a spatial and non-spatial domain [31]. In addition, applied to heat waves, sensitivity measures the capacity of health care systems and population characteristics to respond to changes in weather and climate. Exposure is directly related to the climate hazard (magnitude and variation of the hazard) while adaptation depends on the measures put in place to reduce the adverse impact of the hazard on health and influence the exposure-response relationship in space and time [32]. 

Several studies have already been conducted to assess the vulnerability of populations to climate change, starting with the European EPSON Climate project which has developed a methodology providing a natural hazard impact assessment since 2006 [33]. More specifically, since Susan Cutter’s (et al.) pioneering research on social vulnerability to environmental risks in the United States [34], articles on quantifying vulnerability to extreme heat have followed one another in different ways, identifying physiological vulnerabilities, focusing on the physical state of the individual, and socio-economic vulnerabilities, based on resources and household constitution [30,35,36,37,38].

### 2.1. Physiological Vulnerability

The physiological vulnerability of an individual is defined as those characteristics of the human body that may play a role in the response to heat waves. It can thus be defined as the sum of all physical risk factors that determine whether an individual will experience negative health effects [39]. Some authors also refer to sensitivity to the hazard [34,40]. Indeed, the consequences of a heat wave differ from one category of people to another. According to the literature, the population under 10 years old [34,41,42,43,44,45,46,47,48] and the population over 75 years old are the most vulnerable [30,34,35,41,43,44,45,47,49,50,51,52,53,54]. In addition to age, an individual’s health status itself can increase their vulnerability to heat waves [30,35,41,46,47,52,53,55]. Similarly, it has been shown that individuals with psychiatric disorders or mental illnesses may be more severely affected by high temperatures, including through inadequate hydration [46,50,52]. Finally, some studies highlight the greater vulnerability of women [34,41,43,44,56].

### 2.2. Socio-Economic Vulnerability

The concept of social vulnerability or socio-economic vulnerability is interpreted in different ways in the literature. However, the different resources agree that socio-economic vulnerability is made up of the socio-economic characteristics of a set of people making up a population. For S. Cutter (et al.), it is the product of social inequalities and the characteristics of the community and the built environment [34], or as a measure of the population’s sensitivity to a natural hazard and its ability to cope with it [42]. Borden et al. define vulnerability as social disruption caused by a hazard in a given location [41]. Then, in the 2010s, Holand et al. defined it as the potential loss of human property or capacity [43,57], and Rod et al. as the pre-existing social inequalities in societies that may be affected by a hazard [55], Koks et al. consider social vulnerability as a proxy for wealth, where wealth increases opportunities to prepare for and recover from disasters [45] which suggests a link to notions of resilience and adaptive capacity. Finally, Su et al. described it as the extent to which the social urban system is potentially impacted by external disturbances [48]. In conclusion, social vulnerability is influenced by the social and economic capacities of an individual or community to cope with external threats, and is not based solely on the health status of the individual, unlike physiological vulnerability, as discussed above.

This concept is also not easily measurable since it is not a clearly observable event [58]. However, the different socio-economic vulnerability assessment methods have in common the integration of several socio-economic indicators of different dimensions in order to create a single indicator that is easily understood and applicable by the planners [34,59,60,61,62]. 

### 2.3. Study Area: the Metropolis of the Greater Lyon Area

**Figure 2:**
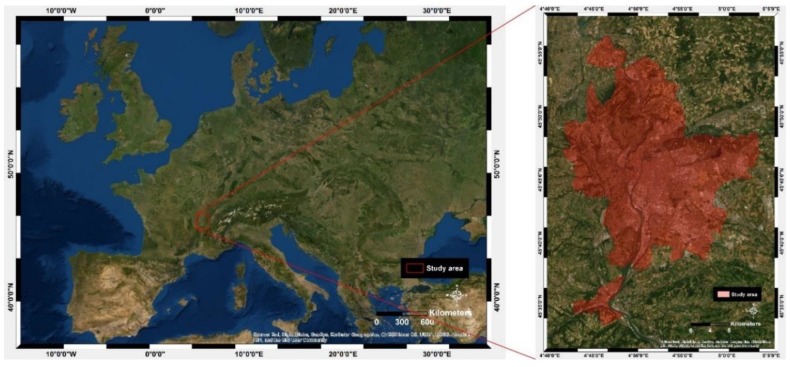
Location of the Metropolis of the Greater Lyon area (source ESRI).

The area of application of this study is the Metropolis of the Greater Lyon. It is a metropolitan area made up of 59 municipalities and has more than 1.3 million inhabitants. It is the second largest metropolitan area in France after Paris, with 2.2 million inhabitants. The Metropolis of the Greater Lyon is located in the south-east of France, at latitudes and longitudes 45°45′35′′ N, 4°50′32′′ E (Figure 2). 

Due to its geographical location, this agglomeration is characterised by a warm temperate climate, influenced by the Mediterranean climate, with high temperatures in spring and summer. Thus, according to Köppen’s classification, the agglomeration is located in Cfa or Cfb, depending on the year. The hottest months are from June to September, with a maximum daily temperature of between 24.6 and 27.7 °C and an average relative humidity of around 55%. According to Mann-Kendall’s trend test [63], since 1921 (beginning of measurements at the Lyon-Bron meteorological station, Météo-France), the annual averages of minimum and maximum temperatures have significantly increased [64,65].

**Figure 3:**
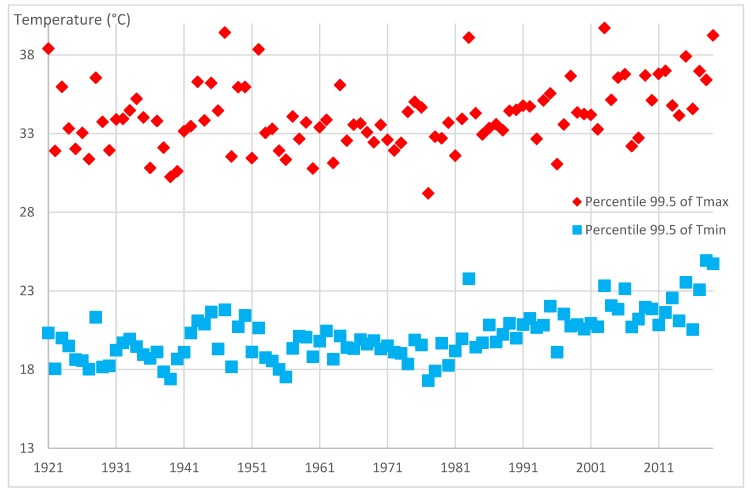
Evolution of the 99.5 percentiles of Tmax and Tmin over the period 1921–2019 (source: Météo-France).

Average increases of 0.20 °C and 0.26 °C, respectively, for the maximum and minimum temperatures per decade have been observed since the beginning of the measurements at the Lyon-Bron station, more precisely between 1921–1930 and 2010–2019. In addition, it is forecast that by 2050 Lyon will have the climate of Canberra (Australia), with an increase in annual temperatures of 1.8 °C compared to the reference period 1971–2000 [66]. The number of summer days, which is defined according to the ETCCDI indices as days with maximum temperatures above 25 °C [67,68], tends to increase over the same period. The number of heat waves has increased sharply since the decade 1994–2003 [23]. Over this period, a heat wave has been identified when the minimum and maximum temperature averaged over three days are above the 99.5 percentiles of their distribution [23]. In addition, and due to the evolution of temperatures, the minimum and maximum threshold temperatures fluctuate each year (Figure 3). 

Thus, there is an increase in these thresholds for both minimum and maximum temperatures. According to the Mann-Kendall test, the maximum temperature threshold values have a Kendall’s tau of 0.23 and the minimum temperature threshold values have a Kendall’s tau of 0.43 for a *p*-value lower than 0.05, for the period 1921–2019. The 99.5 percentile has been selected in this study [23] but there are several indices starting with the Excess Heat Factor [69], which takes the 95 percentile or the Warm Spell Duration Index (WSDI)(WSDI: https://www.climdex.org/learn/indices/) with the 90 percentile.

## 3. Methodologies

### 3.1. The Application of AHP to Assess Physiological Vulnerability

In order to spatialize physiological vulnerability, eight indicators from a review of the literature on the subject influencing a person’s ability to respond to heat wave hazards were selected (Table 1). They are based on age and gender, as well as type of illness, where applicable [35,44,47,49,50,51,52,53,54,70]. These eight vulnerability factors should then be weighted using a method of decision support, as some of them may play a key role in explaining physiological vulnerability.

Methods of decision support are used to facilitate the problems of choosing different alternatives, making decisions or evaluating in complex situations where multiple qualitative and quantitative criteria are involved. They allow several of them to be aggregated with the objective of selecting one or more actions, options or solutions. In this case, the objective is to give priority to different population groups according to their vulnerability to heat waves. Therefore, it is necessary to use a ranking procedure in order to weight them. Several decision support methods exist, each with their advantages and weaknesses. The use of one method rather than another is defined by main parameters such as objectives, ease of use, relevance of assessment, flexibility and time required for implementation and feedback. After an evaluation of these different techniques, our choice turned to pairwise comparison, and more precisely the Analytic Hierarchy Process (AHP) [71]. It allows us to order the alternatives on the basis of either a single criterion, or of different criteria apprehended in their diversity.

The AHP is a theory for measuring criteria in a given situation, based on the derivation of priorities of relative importance from pairwise comparisons of homogeneous alternatives sharing a common attribute [72,73]. This method uses a systems approach (focusing on the functioning of the whole) and a deductive approach (the interrelationship of parts) to structure a complex situation into different elements that can interact with each other, quantify them, and assign values to them relative to their impacts on the whole system. This quantification of the values of the different elements relies on the experience and judgement of a panel of experts to obtain the weights of the heat wave vulnerability functions.

This hierarchical multi-criteria analysis method is thus based on four main steps that will be implemented in the following section, and which will be completed by risk mapping:

- detailed description of the 8 vulnerability factors of the population to characterize, synthesize and decompose the complex situation;
- series of semi-directed interviews with the expert panel where they make binary comparisons of vulnerability factors they appear to have a higher vulnerability to heat waves than other experts.
- validation of the consistency of the experts’ responses and calculation of the weighting of vulnerability factors
- aggregation of responses and calculation of physiological vulnerability to heat waves

The second stage of the procedure is based on semi-directed interviews with the expert panel, which is based on a questionnaire (Table 2). Therefore, the objective is to prioritise the previously selected heatwave vulnerability factors. 

The AHP methodology [72,74] requires prioritisation by binary comparison: a weighting of 1 results in equal vulnerability of the two elements and a weighting of 9 (or 1/9) results in an absolutely higher vulnerability of one function relative to another. Furthermore, in order to retain an expert opinion, the consistency ratio of the health expert’s responses must be less than 0.2 (acceptable judgement—step 3). The physiological vulnerability index is based on the opinion of 35 health professionals, divided into 20% general medical practitioners, 40% pharmacists and 40% nurses. The expert evaluations come from doctors and nurses working in private practice, in hospitals or retirement homes.

The scale of analysis is that of IRIS, the smallest demographic census unit in France. It focuses on between 1800 and 5000 persons. This study has been conducted using the latest INSEE census, which has been made in 2019, for the age and sex criteria. For individuals suffering from chronic or acute pathology and individuals with psychiatric disorders, the Balises Rhône-Alpes database (regional health observatory in Rhône-Alpes) was mobilized, which has been made in 2016. As the latter data are only available at the commune level, an upgrade to the IRIS scale was carried out on a pro-rata basis for the population.

### 3.2. Treatment of Socio-Economic Vulnerability Using PCA

40 selected variables (Table 3) were selected from the scientific literature to characterize socio-economic vulnerability [30,34,41,42,43,45,46,48,55,57]. They are extracted from different databases downloaded from several platforms: Institut national de la statistique et des études économiques (National Institute of Statistics and Economic Studies or INSEE, http://www.insee.fr/fr/accueil ), Observatoire régional de la santé en Rhône-Alpes, Balises Rhône-Alpes (regional health observatory in Rhône-Alpes, http://www.balises-rhone-alpes.org/), Données publiques de l’État français (public data of the French State, Data Gouv https://www.data.gouv.fr/fr/), Open Data de la Métropole de Lyon (Open Data of the Metropolis of the Greater Lyon area, https://data.beta.grandlyon.com/accueil), Notaire de France (https://immobilier.statistiques.notaires.fr/ base-donnees-immobilieres) and Institut national de l’information géographique et forestière (National Institute of Geographic and Forestry Information, IGN, https://geoservices.ign.fr/). When the data for a variable were only present at the commune or district level, they were transposed to the IRIS scale by weighting by population or by area for length variables.

A binary comparison by the AHP method was clearly not possible with a set of 40 variables. The process would have been far too complex and time-consuming for the experts. As an alternative, a PCA with Varimax rotation was used to obtain a socio-economic vulnerability index at the IRIS scale. The various steps involved in the construction of this index are now detailed.

#### 3.2.1. Initial Statistical Tests and Realization of PCA with Varimax Rotation

In the context of this study on the spatialization of socio-economic vulnerability, the objective of using a PCA with Varimax rotation is to extract a set of factors regrouping a selection of the 40 predictors and explaining a maximum of the total variance. Thus, in a first step, all the data of the 40 variables were centred and reduced by the zero-mean normalisation method. This treatment makes it possible to remove the potentially excessive weight of extreme values on the variable in question by generating data independent of the unit or scale chosen [75]. All variables therefore have the same mean and dispersion and can be compared with each other. 

Subsequently, since the variables follow a normal distribution according to the Shapiro Wilk test (applicable to samples with fewer than 5000 observations) [76], a Pearson correlation matrix is used to detect collinear data. For each pair of indicators with a |r| > 0.7, one is suppressed. The value of the variance inflation factor (VIF) is also controlled and variables with a score greater than 5 are also removed [75]. 

A second statistical step in the selection of variables is performed using the Kaiser-Meyer-Olkin test (KMO) [77]. It is used to verify that, once the linear effect of the other items has been controlled for, the partial correlations of each pair of items are low, which would confirm the presence of latent variables linking the items together. In other words, it indicates the extent to which the set of variables selected is a coherent set. Here, variables with a KMO < 0.6 have been removed [75].

Then, the statistical verification is based on several tests. First, Bartlett’s sphericity test validates or invalidates the hypothesis that the variables are not significantly correlated [78]. In the case of our set of variables, Bartlett’s sphericity test indicates that the variables are not significantly correlated.

Subsequently, to select the relevant variables and group them into factors (dimensions), a Principal Component Analysis (PCA) with Varimax rotation was used [79]. PCA makes it possible to search for a solution to the total variance of the measured variables. PCA groups variables into factors where only those with eigenvalues greater than 1 are retained.

Finally, statistical testing is continued by analyzing the value of Cronbach’s alpha coefficient. This statistical test is used in particular in psychometrics to measure the internal consistency (or redundancy of information) of the questions asked during a test. An alpha value around 0.7 is considered to be satisfactory [80]. Here, Cronbach’s mean alpha coefficient is 0.72.

#### 3.2.2. The weighting of the Final Variables Selected

Following the selection of the variables after the PCA into different components, the final step to obtain the socio-economic vulnerability index is their aggregation. Variables that increase socio-economic vulnerability are added together, such as the rate of persons over 75 years old, and those that decrease it, such as persons with higher education, are subtracted. Then the variables within the factors can be weighted or unweighted. For example, Cutter et al. (2003) [34] make “no a priori assumption about the importance of each factor in the overall sum”, seeing them “as having an equal contribution to the county’s overall vulnerability”, due to “an the absence of a defensible method for assigning weights”. On the contrary, some other authors, such as Madrigana et al. [30], Holand et al. [43], Holand and Lujala [57] or Wolf and McGregor [81] weigh the factors retained by their variance and combine them to obtain a final score. We opted for this method, weighting by the value of the correlation coefficient determined by the Varimax rotation. In the end, the combination of the different components constitutes the final index.

## 4. Results

### 4.1. The Physiological Vulnerability

**Figure 4:**
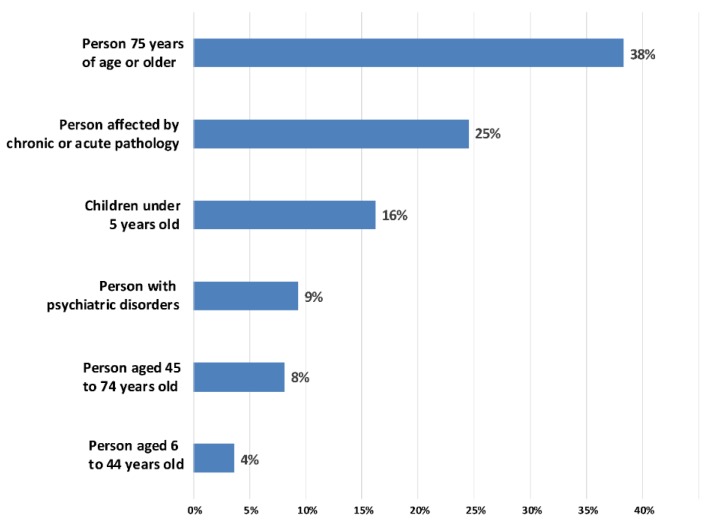

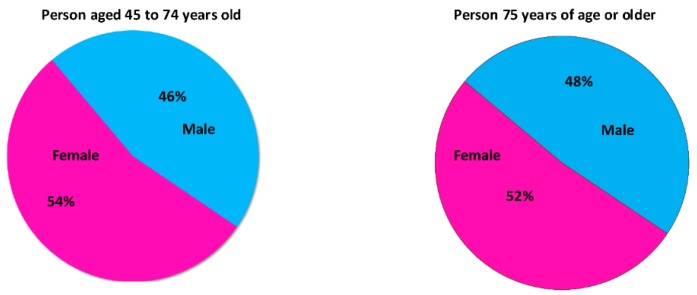
Relative physiological vulnerabilities for different categories of the population to heat waves.

The results of the AHP, based on the binary comparisons of the expert panel, make it possible to prioritize the vulnerabilities of the previously identified population categories (Table 1 and Figure 4). As expected, and in agreement with the literature, people aged 75 years and over is the population category most vulnerable to heat waves (38%). Next come people suffering from chronic or acute pathologies (25%), followed by children under 5 years of age (16%). 

It is interesting to note that the gender of the individual is only slightly highlighted by the experts interviewed. Indeed, women aged 45–74 are only slightly more vulnerable (54%) than men in the same age group, such as, according to the experts questioned, women aged 75 and over are just as sensitive (52%) as men (48%). Therefore, it is clear that in the case of this study, gender is not a predominant variable, particularly in relation to older age or people with chronic or acute pathologies.

**Figure 5:**
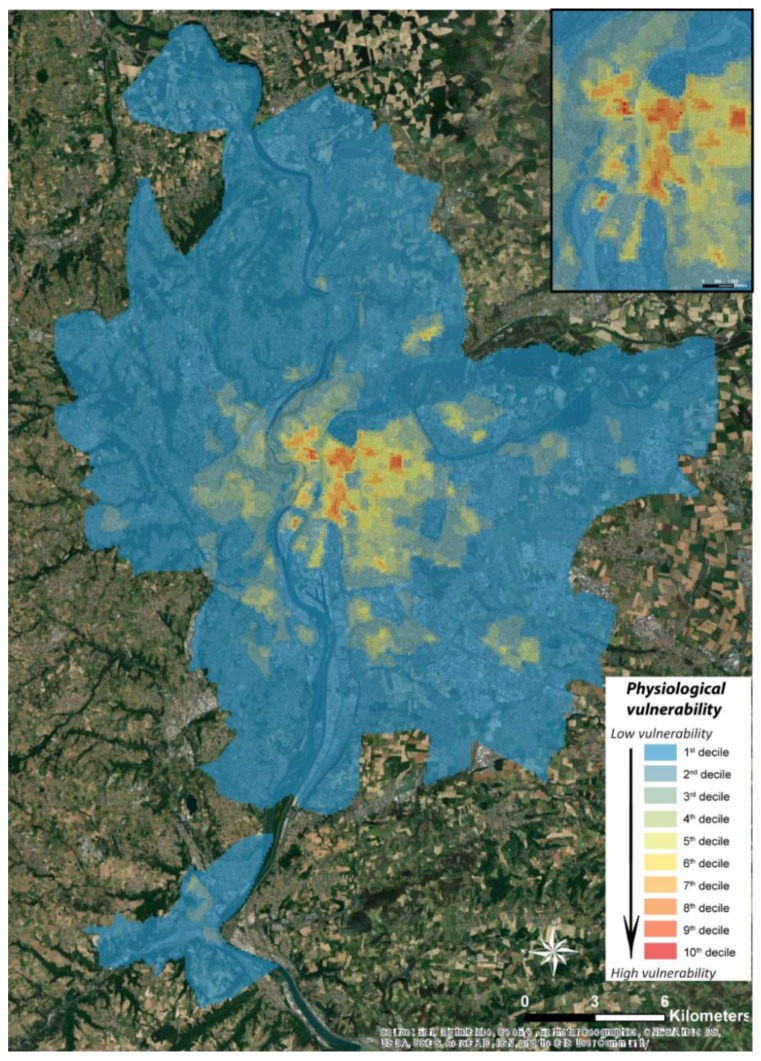
Spatial Representation of Physiological Vulnerability to Heat Waves (discretization by deciles)—Zoom on the center of Lyon in top right.

The highest vulnerability rates are obtained in the centre of Lyon, i.e., in the 1st district, the north of the 2nd as well as the 7th district, the south of the 4th and 6th district, but also in Villeurbanne (Figure 5). 

A small part of the municipalities of Vaulx-en-Velin (Le Mas du Taureau) and Rillieux-la-Pape are also heavily impacted with a vulnerability index of 0.5. Also, to be considered, the commune of Vénissieux (Les Minguettes) and the sector of the 8th district of Lyon which have a vulnerability index around 0.4. The sectors with the lowest vulnerability are located on the periphery of the Metropolis of the Greater Lyon as well as to the north of the 6th district and to the south of the 7th district, with a rate very close to zero vulnerability.

### 4.2. The Socio-Economic Vulnerability

According to the various statistical tests for evaluating the collinearity or consistency of the dataset, 26 variables were retained. After performing Principal Component Analysis (PCA) with Varimax rotation, 71% of the total variance is explained by six factors (Table 4). 

Each factor contributes to increasing or decreasing the socio-economic vulnerability of populations to heat waves through the variables they contain:

- Factor No. 1 groups together variables representative of the socio-economic defaults of the metropolis: people with low-skilled jobs, people without higher education, people with higher education, unemployment rate, percentage of social housing, average annual household wage, percentage of children under 5 years old and poverty rate.
- Factor No. 2 tends to include characteristics of physical defaults with the number of persons undergoing psychotropic drug treatment per capita, the percentage of persons receiving the disabled adult allowance and the percentage of the population who died before the age of 65, supplemented by the number of births per capita.
- Factor No. 3 highlights the vulnerability of the elderly with the variables relating to the percentage of retired persons and the percentage of persons aged 65 and over and the percentage of persons without a diploma.
- Factor No. 4 is considered an indicator of territorial development since it includes the mortality rate, the distance from the nearest home to the nearest hospital, and the number of beds available per hospital.
- Factor No. 5 focuses on the vulnerability of the female population by grouping together the percentage of women in the total population of metropolitan Lyon and the percentage of working women.
- Finally, the last factor gathers the health-related variables, with the number of medical professions per inhabitant and the number of health establishments (public or private) per inhabitant.

Factors 1, 2, 3 and 5 contribute in their totality to increasing the socio-economic vulnerability of populations to heat waves, while factors 4 and 6 participate in reducing this vulnerability on the territory of the Metropolis of the Greater Lyon.

**Figure 6:**
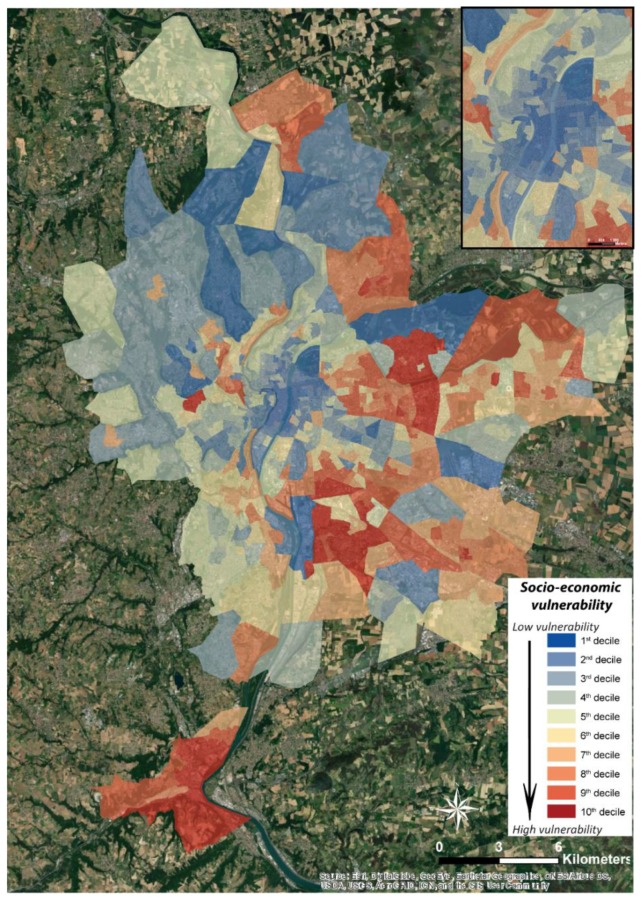
Spatial representation of the socio-economic vulnerability of Lyon’s population to heat waves—(discretization by deciles)—zoom on the center of Lyon in top right.

Similar to physiological vulnerability, these results are mapped on the territory of the metropolis of Lyon using a GIS (Figure 6), with values ranging from 0 (zero vulnerability) to 1 (maximum vulnerability). From the outset, it appears that the most vulnerable areas from a socio-economic point of view are located on the periphery. These results are very different from the most vulnerable areas identified in the physiological vulnerability analysis, which are located in the centre of the agglomeration (Figure 4). Indeed, the most vulnerable sectors of the Lyon metropolitan area are located in the two communes in the south of the urban area (Givors and Grigny) with values approaching 0.7, but also and above all in the south-east and east, respectively in the communes of Vénissieux and Vaulx-en-Velin, with a vulnerability index that reaches the maximum value (Anatole France IRIS) and 0.83 respectively. In addition, some communes in the north and north-east of the agglomeration have values of around 0.6 in Meyzieu, Décines-Charpieu, Rillieux-la-Pape and Neuville-sur-Saône. An IRIS located in the west of Lyon is attracting attention. Indeed, with a socio-economic vulnerability index of 0.73, this sector of Tassin-la-demi-Lune stands out clearly from its immediate neighbours who do not show high values. As for the centre of the agglomeration, it stands out by extremely low vulnerability values, most of the time lower than 0.2.

## 5. Discussion

### 5.1. A Global Socio-Economic Vulnerability to Be Enhanced Using the Factors

**Figure 7:**
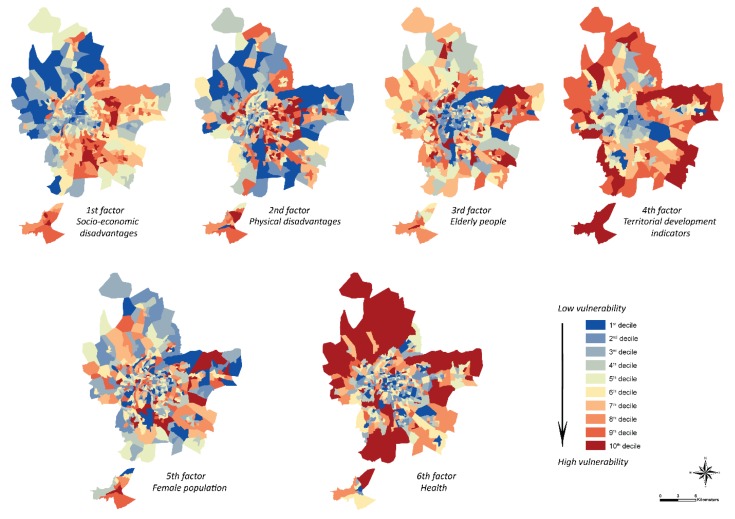
Spatial representation of socio-economic vulnerability in the Metropolis of Lyon to heat waves by factors—discretization by equal intervals.

Global socio-economic vulnerability is a multidimensional index that helps identify the characteristics and experiences of a community that enable them to cope with and recover from environmental hazards [34]. However, it is important to relativize this global vulnerability index, whose spatial distribution can prove to be very heterogeneous when we look at its component factors [82]. Indeed, depending on the type of vulnerability considered and highlighted by the 6 factors of the PCA with Varimax rotation, the territories concerned can be radically different (Figure 7). For example, for municipalities located to the east and south of the metropolis, factors 1 “socio-economic defaults”, 2 “physical defaults”, 4 “territorial development indicators” and 5 “female population” are the most influential. Respectively, this corresponds to variables such as a high number of social housing units, a high poverty rate, a high number of people with low-skilled jobs and a high number of people without qualifications, a high number of premature births and deaths, a high mortality rate and few working women. In the same way, the socio-economic vulnerability of the municipalities in the west and north of Lyon appears to be important for factors 3, “elderly people”, 4 “territorial development indicators”, 5 “female population” and 6 “health”. This would correspond respectively more to variables such as a high number of pensioners, a high distance to the nearest hospitals, few active women and a low density of medical professions.

These diversities of socio-economic vulnerabilities are explained by territorial logics particular to each of the territories under consideration and it is therefore advisable not to stop at the overall result of socio-economic vulnerability but to continue the analysis by focusing on the various factors that make it up. Indeed, in order to combat the risks associated with high temperatures, public policies will have to be adapted locally according to the people at risk considered and their types of vulnerability, particularly since the resources devoted to this are not inexhaustible. For example, the overall socio-economic vulnerability map shows us that the most vulnerable sectors are located in the north, northeast, east and remote southern municipalities. However, if communication campaigns are to be carried out to make the elderly aware of the dangers of high temperatures, there is a real need to focus on the third component of the PCA, which highlights the west of the agglomeration as a sector of high vulnerability, something that the distribution of overall vulnerability did not at first appear to suggest.

### 5.2. Physiological and Socioeconomic Vulnerabilities That Are Spatially very Divergent

The comparison of the spatial distributions of physiological and socio-economic vulnerabilities (Figure 5 and Figure 6) shows a significant asymmetry. Indeed, physiological vulnerability follows a decreasing gradient from the centre to the periphery, while socio-economic vulnerability highlights the eastern part of the agglomeration, which extends from north to south. This is confirmed by the Spearman coefficient (non-normal distributions of variables) of −0.015 between these two types of vulnerability (Table 5).

This is due to the two types of vulnerabilities being radically different in their approaches, concepts and practices, and not only to the types of variables used. In fact, the methodology and subsequent results of socio-economic vulnerability highlight the most disadvantaged sectors, whereas the methodology of physiological vulnerability takes more account of the residential density of the population, particularly individuals aged 6 to 44 (Table 1). Although this population group accounts for only 8% in the expression of the physiological vulnerability effect, its weight within the total population of the metropolis shows this decreasing gradient from the centre to the periphery. This is confirmed when we consider the Spearman’s coefficient between this vulnerability and the residential density of the agglomeration, which is 0.97 (Table 5). Thus, the distribution of physiological vulnerability is very similar to that of residential density. This is not necessarily a limitation of this method, as it is capable of associating the residential density of the population in the strict sense with their various vulnerability factors. From our point of view, the overall socio-economic vulnerability map should be interpreted even more cautiously. In fact, it shows a crown of greater vulnerability on the periphery, to the east of the agglomeration, which could lead decision-makers and managers to believe that the center of the agglomeration is practically not vulnerable to high temperatures, whereas the majority of the metropolis’ population is concentrated there. Mapping representations of the six factors of socio-economic vulnerability (Figure 7) would be more useful, by precisely focusing on the sectors at risk, according to their types of vulnerability, in order to best adapt public policies according to the populations and their receptiveness.

### 5.3. A Perception of Physiological Vulnerability That Differs According to the Training of Experts

**Figure 8:**
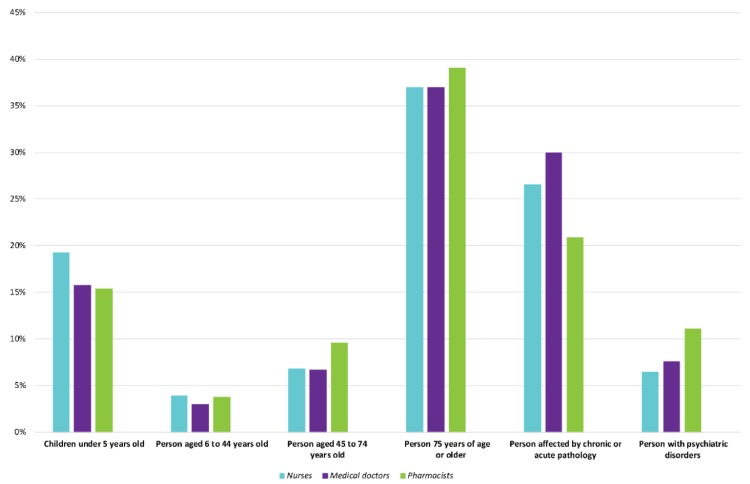
Weights of Vulnerability Factors by Medical Professions.

The Physiological Vulnerability Index, obtained using the AHP, is based on the opinions of health professionals from different occupational categories: physician, nurse and pharmacist. Depending on the profession, a difference in the prioritization of the criteria is observed (Figure 8). 

As a result, this diversity of opinion may have an impact on the final weighting of the variables. For example, the weighting assigned to persons with chronic or acute pathologies varies from 21% from the physicians’ point of view to 30% from the pharmacists’ point of view. Thus, this ranking is based on a geometric mean of the assumptions of importance between the criteria granted by the panel of experts questioned. This way of clustering opinions has been shown to be the most relevant [83]. It should be noted, however, that the differences in weighting between the different occupations are minor and that the overall ranking is not questioned, with people over 75 years of age and individuals with chronic or acute conditions being designated as the most vulnerable to extreme heat. On the contrary, if major differences in judgements had appeared between occupations or between experts in the same occupation, other methods such as those of Ramanathan and Ganesh or the Fuzzy Delphi method [84] could have been used [85,86]. Finally, while the AHP has already demonstrated its potential in this type of issue, other methods have also been found to be relevant in the aggregation of judgments, such as the individual judgments (AIJ) and the aggregation of individual priorities (AIP) [85].

Another limitation to be raised in the process of collecting expert opinions for the assessment of physiological vulnerability is the lack of expert opinions from psychiatric centers. Indeed, according to the health professionals consulted, this category of person is more sensitive to heat waves than those without psychiatric problems. Consultations with psychiatric experts could have confirmed these opinions.

## 6. Conclusions

The aim of this study was to spatialize two distinct types of vulnerabilities according to two drastically different methodologies, in order to confront them and to locate the sectors of the Lyon metropolis that could be the most vulnerable to high temperatures. However, the definitions and ways of understanding the notion of vulnerability are plethoric, as are the operating methods that can be used to spatialize them. Thus, after a review of the literature on the subject, our choice fell on physiological and socio-economic vulnerabilities, explored respectively using AHP and a PCA with Varimax rotation. The application of these two methods yielded relatively dissimilar results from a spatial point of view. Indeed, the area of greatest physiological vulnerability is located in the center of the agglomeration, while the most socio-economically unfavourable areas are mostly located on the periphery. This might seem contradictory, even quite confusing for territorial engineers who are not used to working with such methods. It is therefore appropriate to return to the bases of these two vulnerabilities, which do not aim to identify the same types of vulnerabilities, and to the ways of apprehending them. Indeed, according to the 35 experts interviewed, the physiological vulnerability to heat waves of the population of Lyon is directly linked to people over 75 years of age, people with chronic or acute pathologies or children under 5 years of age. However, as it is necessary to include populations aged 6 to 75 in binary comparisons, the latter, due to their high densities in the center of the metropolis, explain this concentric organization of vulnerability. Conversely, a crown of greater socio-economic vulnerability appears in the east of the metropolis, notably because the residential density of the population is not retained by the PCA. The latter makes it possible to explain 71% of the variance using 6 factors. This socio-economic vulnerability also needs to be analyzed in terms of its various components. These 6 factors selected contain variables relating to the socio-economic disadvantages of the territory, the physical disadvantages, the elderly, the level of territorial development, the female population and health care. Thus, the most vulnerable areas of the study area are explained in particular by a strong presence of social housing, a high rate of poverty, a high number of people with low-skilled jobs or without qualifications and a high birth and premature death rate. The presence of the female population is also a determining and dominant variable in establishing a high rate of socio-economic vulnerability to heat waves. The use of a specific vulnerability should therefore be selected according to public policy objectives.

Furthermore, as the metropolis of Lyon has made considerable economic progress over the last ten years, it would be interesting to carry out a spatio-temporal analysis of physiological and socio-ecological vulnerabilities and to characterize their evolution [42]. It would also be very useful for decision-makers and local politicians to compare the different vulnerabilities of the territory to air temperatures or thermal comfort indices during heat waves. A characterization study of thermal gradients is already underway in the Metropolis of the Greater Lyon. The results will make it possible to locate the sectors prone to urban overheating, but more importantly the areas to be targeted for a reduction in the associated risk, by comparing them with the results of this study of vulnerabilities. On these vulnerable areas that have been identified, the metropolitan authority can carry out planning actions in favour of reducing people’s sensitivity to heat waves, such as implanting trees to create shade, both on public spaces and on buildings [87]. In addition, these heat wave vulnerability evaluation methods are replicable in any city as they require only access to appropriate databases, expert health advice and basic computer facilities with the installation of free and open source geographic information system software (such as QGis) and statistical processing (such as R).

## Figures and Tables

**Table 1:** Variables used in the assessment of physiological vulnerability to heat wave hazard.

Selected Variables	Effect on the Vulnerability	References
Children under 5 years old	Increase	[44,47]
Person aged 6 to 44 years old	Decrease	[49,50]
Person aged 45 to 74 years old	Decrease	[50]
Person 75 years old or older	Increase	[35,44,47,49,50,51,52,53,54]
Sex for 45–74 years olds	The greater the number of women, the more vulnerable they are	[44]
Sex for over 75 years olds	The greater the number of women, the more vulnerable they are	[44]
Person affected by chronic or acute pathology	Increase	[35,47,52,53]
Person with psychiatric disorders	Increase	[50,52]

**Table 2:** Questionnaire completed by the health experts interviewed.

Children Under 5 Years Old	9	8	7	6	5	4	3	2	1	2	3	4	5	6	7	8	9	Person Aged 6 to 44 Years Old
Children under 5 years old	9	8	7	6	5	4	3	2	1	2	3	4	5	6	7	8	9	Person aged 45 to 74 years old
Children under 5 years old	9	8	7	6	5	4	3	2	1	2	3	4	5	6	7	8	9	Person 75 years of age or older
Children under 5 years old	9	8	7	6	5	4	3	2	1	2	3	4	5	6	7	8	9	Person affected by chronic or acute pathology
Children under 5 years old	9	8	7	6	5	4	3	2	1	2	3	4	5	6	7	8	9	Person with psychiatric disorders
Person aged 6 to 44 years old	9	8	7	6	5	4	3	2	1	2	3	4	5	6	7	8	9	Person aged 45 to 74 years old
Person aged 6 to 44 years old	9	8	7	6	5	4	3	2	1	2	3	4	5	6	7	8	9	Person 75 years of age or older
Person aged 6 to 44 years old	9	8	7	6	5	4	3	2	1	2	3	4	5	6	7	8	9	Person affected by chronic or acute pathology
Person aged 6 to 44 years old	9	8	7	6	5	4	3	2	1	2	3	4	5	6	7	8	9	Person with psychiatric disorders
Person aged 6 to 44 years old	9	8	7	6	5	4	3	2	1	2	3	4	5	6	7	8	9	Person 75 years of age or older
Person aged 45 to 74 years old	9	8	7	6	5	4	3	2	1	2	3	4	5	6	7	8	9	Person affected by chronic or acute pathology
Person aged 45 to 74 years old	9	8	7	6	5	4	3	2	1	2	3	4	5	6	7	8	9	Person with psychiatric disorders
Person 75 years of age or older	9	8	7	6	5	4	3	2	1	2	3	4	5	6	7	8	9	Person affected by chronic or acute pathology
Person 75 years of age or older	9	8	7	6	5	4	3	2	1	2	3	4	5	6	7	8	9	Person with psychiatric disorders
Person affected by chronic or acute pathology	9	8	7	6	5	4	3	2	1	2	3	4	5	6	7	8	9	Person with psychiatric disorders
Person aged 45 to 74 years old																		
Male	9	8	7	6	5	4	3	2	1	2	3	4	5	6	7	8	9	Female
Person 75 years of age or older																		
Male	9	8	7	6	5	4	3	2	1	2	3	4	5	6	7	8	9	Female

**Table 3:** Variables selected in the assessment of socio-economic vulnerability to heatwave hazard.

Selected Variables	Author
Ratio of the number of births domiciled at the mother’s home	[34,41,42,43,46]
Average age of the population	[41,42,43]
% of the population between the ages of 18 and 64	[55]
% of population under 5 years old	[34,41,43,45,48,57]
% of population over 65 years old	[30,34,41,43,45]
Ratio of females to males	[43,57]
% of female population	[34,41,43,46]
number of employed persons with low-skilled jobs between 15–64 years old	[34,43,57]
Poverty rate of the entire population	[34,41,42]
Average age of principal residences over the period 1900 to 2009,	[41,43,46]
% of apartment type principal residents built between 1990 and 2009	[57]
% population living in low-rent housing	[43]
% population in main residence occupied free of charge	[34]
% of precarious housing	[34]
% of employed women between 15–64 years old	[34,41,42]
% of employment in the population between 15 and 64 years old	[34,41]
% of the employed population between 15–64 years old working in farming	[41,55,57]
Unemployment rate of the employed population between 15 and 64 years old	[34,41,48,57]
Unemployment rate of employed women between 15 and 64 years old	/
% employed population with low-skilled jobs	[34,43,57]
% of retired people in 2012	/
% out-of-school population over 15 with no higher education qualification	[34,41,42,46,57]
% population over 15 years out of school with no certificate or diploma	/
% out-of-school population over 15 years old with long-term education at tertiary institutions	[57]
% out-of-school population over 15 years old with higher education	/
Number of medical professions in 2014 per capita	[41,43,57]
Number of health institutions of all types (private or public)	[34,41,42]
Average annual salary in euros	[34,41,42,43,45,55,57]
Mortality rate (all causes) per 1000 inhabitants	[55]
Number of premature deaths from all causes (before the 65 years old) per capita	[43,55]
Number of new long-term care (LTC) admissions per capita	[30,55]
Number of people receiving adult disabled benefit (ADB) per inhabitant	[43,57]
Median household income in euros	[55,57]
Number of people on psychotropic treatment per capita	[46]
Number of hospital places (short or long hospitalisation) per 1000 inhabitants	[41,46]
% of people suffering from psychiatric disorders (Full-time inpatient active file) in 2012	[46]
Number of psychiatric hospital places per 1000 inhabitants	/
Number of care places per 1000 inhabitants	[30]
Proportion of social housing (%)	[30]
Length in kilometres from a hospital by isochrones	[43,57]

**Table 4:** Factors and variables retained after rotational PCA Varimax.

*Factors after PCA with Varimax Rotating*	*Variables*	*Increases (+) or Decreases (−) Socio-Economic Vulnerability*	*Details*		*Weighting*
*Socio-economic disadvantages (+)*	Low-skilled worker	High concentration (+); low concentration (−)	People working in low-skilled jobs can be severely impacted by a heat wave, often working in extreme conditions.		0.92
	No graduate student	High concentration (+); low concentration (−)	People without higher education may not be aware of preventive actions against heat waves.		0.87
	Person with university education	High concentration (−); low concentration (+)	People with higher education may have a greater knowledge of preventive measures to be taken in the event of heat waves.		0.79
	Unemployment rate	High (+); Low (−)	People affected by unemployment do not necessarily have the financial means and appropriate housing to protect themselves from these extreme climatic conditions.		0.76
	Person in social housing	High concentration (+); low concentration (−)	Social housing is more obsolete, often poorly insulated and without centralized air conditioning.		0.73
	Average annual income	High (−); Low (+)	The higher a household’s average annual income, the more it will have the financial means to protect itself from these unpredicted and extreme weather conditions.		0.69
	Person 5 years old or younger	High concentration (+); low concentration (−)	Children under 5 years of age are less physically resistant to extreme heat (more rapidly dehydrated).		0.66
	Poverty rate	High (+); Low (−)	People living below the poverty level do not have sufficient income funds to be able to cope with rising temperatures or to protect themselves from a heat wave.		0.60
	The economic disadvantages of a population, household or individual contribute to increasing their vulnerability to a heat wave.				
*Physical disadvantages (+)*	Birth rate	High (+); Low (−)	Many births may represent increasing family size and thus financial limitations, often with the outsourcing of care for newborns, requiring families to combine their responsibilities with the needs of the family.		0.92
	Number of treatments with psychotropic medications	High concentration (+); low concentration (−)	People taking psychotropic medications are less physically resistant to extreme heat.		0.92
	Number of people receiving an Adult Disability Benefit (ADB)	High contribution (+); Low contribution (−)	People receiving an ADB who are dependent on services		0.87
	Number of premature deaths	High concentration (+); low concentration (−)	The number of premature deaths is associated with poor health and often low income and therefore low physical resistance during a heat wave.		0.84
	**People with physical disavantages are therefore more vulnerable to heat waves.**				
*Elderly people (+)*	Pensioners	High contribution (+); Low contribution (−)	Pensioners are people of advanced age, physically less resistant to high temperatures.		0.83
	People over 65 years old	High contribution (+); Low contribution (−)	People over the age of 65 are less physically resistant to extreme heat (more rapidly dehydrated).		0.79
	Person without a certificate	High contribution (+); Low contribution (−)	People without certificates may not be aware of preventive measures to cope with heat waves.		0.66
	**Elderly people are less physically resistant to extreme heat (more rapidly dehydrated).**				
*Territorial development indicator (−)*	Mortality rate	High (+); Low (−)	A high mortality rate is associated with deteriorated health and therefore low physical resistance of the populations living within this space during a heat wave.		0.73
	Distance to hospital	High (+); Low (−)	A high distance reduces the ability to mobilize sufficient resources in a reasonable amount of time.		0.71
	Number of beds in hospital	High (−); Low (+)	A low number of hospital beds reduces the ability to mobilize sufficient resources in a reasonable period of time to help people cope with the impact of a heat wave.		0.68
	**High territorial development can help reduce the vulnerability of the population to heat waves.**				
*Female population (+)*	Female person	High contribution (+); Low contribution (−)	Women have more physical defaults to cope with the effects of high heat.		0.77
	Non-working woman	High contribution (+); Low contribution (−)	Working women may be less vulnerable than non-working women because they will have more financial resources to prevent heat waves.		0.33
	**Women are generally more vulnerable to the effects of a heat wave.**				
*Health (−)*	Number of medical professions	Numerous (−); not many (+)	A high number of medical professions contributes to increasing the capacity to mobilize sufficient resources in a reasonable time to help populations cope with the effects of a heat wave.	0.77	
	Number of health establishments	Numerous (−); not many (+)	A high number of health establishments contributes to increasing the capacity to mobilize sufficient resources in a reasonable time to help populations cope with the effects of a heat wave.	0.76	
	Rapid and dense health interventions can help reduce people’s vulnerability to heat waves.				

**Table 5:** Spearman coefficient between vulnerabilities, components and residential density (inhab./km^2^).

	Physio	Factor 1	Factor 2	Factor 3	Factor 4	Factor 5	Factor 6	Res. Density
Socio-eco	−0.015	0.886	0.554	0.327	0.445	0.266	0.056	−0.036
Physio	1	−0.036	0.338	−0.302	−0.397	0.084	−0.469	0.970
